# The first species of *Aplastodiscus* endemic to the Brazilian Cerrado (Anura, Hylidae)

**DOI:** 10.3897/zookeys.642.10401

**Published:** 2017-01-03

**Authors:** Bianca V. M. Berneck, Ariovaldo A. Giaretta, Reuber A. Brandão, Carlos A. G. Cruz, Célio F. B. Haddad

**Affiliations:** 1 Departamento de Zoologia, Instituto de Biociências, UNESP, Universidade Estadual Paulista, Campus de Rio Claro, São Paulo, Brazil; 2 Laboratório de Taxonomia, Sistemática e Ecologia de Anuros Neotropicais. Faculdade de Ciências Integradas do Pontal, Universidade Federal de Uberlândia, UFU, Ituiutaba, Minas Gerais, Brazil; 3 Laboratório de Fauna e Unidades de Conservação, Departamento de Engenharia Florestal, Universidade de Brasília, Brasília, Brazil; 4 Departamento de Vertebrados, Museu Nacional, Universidade Federal do Rio de Janeiro, Quinta da Boa Vista, Rio de Janeiro, Rio de Janeiro, Brazil

**Keywords:** Aplastodiscus lutzorum sp. n., Cophomantinae, new species, integrative taxonomy

## Abstract

The genus *Aplastodiscus* includes 14 nominal species in four monophyletic groups with occurrence in the Atlantic Forest and Brazilian Cerrado (Brazilian Savanna) of South America. A recent study reviewed the taxonomy and phylogenetic relationships of the genus and suggested a third species for the *Aplastodiscus perviridis* Group. Herein, on the basis of morphology and advertisement call, we describe this species and test its monophyly. The new species is the only *Aplastodiscus* with endemic occurrence in the Cerrado Biome. In addition, its geographical distribution and conservation status are discussed.

## Introduction

The genus *Aplastodiscus* includes 14 nominal species in four monophyletic groups (Berneck et al. 2016) with occurrence mainly in Atlantic Forest in Brazil and Argentina (Frost 2016). The species are stream-breeding treefrogs, usually of green color and medium size. The *Aplastodiscus perviridis* species Group includes *Aplastodiscus perviridis* Lutz 1950 and *Aplastodiscus cochranae* (Mertens 1952), which share, among other characters, bicolored irises, no webbing between toes I and II, and reduced webbing among their remaining toes (Garcia et al. 2001).

Berneck et al. (2016) recently reviewed the taxonomy and phylogenetic relationships of all *Aplastodiscus* species and suggested a third species for the *Aplastodiscus perviridis* Group, the “*Aplastodiscus* sp. 3”. This species was previously called *Aplastodiscus perviridis* by the previous authors (Garcia et al. 2001; Bastos et al. 2003; Morais et al. 2012; Valdujo et al. 2012). Herein, on the basis of morphology and advertisement call, this species is described as new, and its monophyly tested. In addition, its geographical distribution and conservation status are discussed.

## Materials and methods

### Descriptions of adults and their calls

The following measurements follow Duellman (1970):

SVL
 snout-vent length 


HL
 head length 


HW
 head width 


ED
 eye diameter 


TD
 tympanum diameter 


END
 eye-nostril distance 


IOD
 interocular distance 


THL
 thigh length 


FL
 foot length; including tarsus 


However, the tibia length (TBL) follows Heyer et al. (1990). Measurements are in millimeters and were taken with a digital caliper and, except for SVL, HL, HD, THL, and TBL, under a stereomicroscope. The webbing formula follows Savage and Heyer (1967) and Myers and Duellman (1982). Adult males were collected while calling and/or recognized by the presence of vocal slits.

The adult specimens are housed in the following Brazilian collections: Célio F. B. Haddad collection (CFBH) at the Universidade Estadual Paulista, Rio Claro, SP; Coleção Herpetológica da Universidade de Brasília (CHUNB) at the Universidade de Brasília, Brasília, DF; Museu de Ciências Naturais da Pontifícia Universidade Católica de Minas Gerais (MCN-AM), Belo Horizonte, MG; and Coleção de Anuros da Universidade Federal de Uberlândia (AAG-UFU), Uberlândia, MG.

Males of the new species were recorded in the Municipality of Alto Paraíso de Goiás, Goiás State (N = 6) and Brasília, Distrito Federal. For comparative purposes, males of *Aplastodiscus perviridis* were recorded at the type-locality (N = 5), in Serra da Bocaina, São José do Barreiro, São Paulo State. Calls were recorded with a Marantz PMD 671, a Boss BR-864 (both with a Sennheiser ME67/K6 microphone) or a MicroTrack (ME66/K6 microphone), all set at 44.1 kHz and 16-bit resolution. Calls were recorded from 50 to 150 cm from calling males, and 10 to 15 calls were analyzed for each male. Acoustic variables were analyzed with RAVEN PRO 1.5, 64-bit version (Bioacoustics Research Program 2014); terminology used for call features were according to Raven’s manual (Charif et al. 2010). A 500 Hz high pass filter was applied prior to call analyses and figuring to reduce wind noise interference. Sound figures were obtained with the SEEWAVE 1.6.4 (Sueur et al. 2008) R package (R Development Core Team 2012, v. 2.15.1), settings used were the Hanning window, 85% overlap, and 256 points resolution. Measured call parameters were: 1) call duration (CD), 2) peak of dominant frequency (PDF), 3) lower dominant frequency (LDF), 4) higher dominant frequency (HDF), 5) time to frequency peak (TFP) (expressed as % of call duration). All calls used in descriptions are housed at the AAG-UFU collection (Suppl. material 1, Table 1).

**Table 1.** Acoustic variables of the advertisement call of topotypes *Aplastodiscus perviridis* and *Aplastodiscus lutzorum* sp. n. n = number of recorded males.

Call Features	*Aplastodiscus lutzorum* sp. n. (n = 12) Range Mean (SD)		*Aplastodiscus perviridis* (n = 5) Range Mean (SD)	
Call Duration (seconds)	0.26–0.40	0.32 (0.05)	0.12–0.15	0.13 (0.01)
Higher Frequency (kHz)	2334–2647	2468 (97)	2419–2750	2519 (135)
Lower Frequency (kHz)	1494–1732	1595 (76)	1587–1806	1690 (82)
Dominant Peak (kHz)	1884–2156	2027 (79)	1981–2153	2078 (66)
Time to Frequency Peak (%)	49–70	61 (7)	23–38	34 (6)
Air temperature range	19–22 °C		16–19 °C	
Record hour	20:00–22:00 h		20:30–21:00 h	

### Laboratory protocols and genetic distance calculation

The extraction of DNA was carried out using ethanol-preserved tissues and the DNeasy isolation kit (Qiagen, Valencia, CA, USA). We carried out DNA amplification in a 25 µL volume reaction using master mix Fermentas Taq Polymerase and reagents. The Polymerase chain reactions (PCR) included an initial denaturing step of 30s at 94 °C, followed by 35 cycles of amplification with a final extension step at 72 °C for 6 min. The products of PCR were sent for sequencing to Macrogen, South Korea. We sequenced DNA fragments in both directions to minimize potential errors. The chromatograms were read and edited using SEQUENCHER 3.0 (Gene Codes, Ann Arbor, MI, USA) and complete sequences were edited with MEGA 6.06 (Tamura et al. 2013). The distance estimations of genetic p-distances were calculated in MEGA 6.06 for the regions delimited by the primers 16sAR (Palumbi et al. 1991), Wilk2 (Wilkinson et al. 1996), and COI (Jungfer et al. 2013), considering d:transitions + transversions, uniform rates among sites, and gaps/missing data as complete deletion. A list of vouchers, GenBank accession numbers, and locality data is available in Suppl. material 2.

### Phylogenetic analysis and taxon sampling

Berneck et al. (2016) studied *Aplastodiscus* in a wider context and consequently included only one specimen of the species described here. Therefore, we carried out a reduced phylogenetic analysis that included all terminals of the *Aplastodiscus perviridis* Group analyzed by Berneck et al. (2016) and four specimens of the species described here. As outgroups, we included two terminals of the *Aplastodiscus albosignatus* Group and two of the *Aplastodiscus albofrenatus* Group, rooting the tree in the *Aplastodiscus sibilatus* Group (see Berneck et al. 2016). The dataset used for the analysis were the fragments delimited by the primers 16sAR, Wilk2, and COI forward and reverse.

Sequence alignments were performed in Clustawl (Thompson et al. 1994) under MEGA 6.06. For the phylogenetic analysis T.N.T Willi Hennig Society Edition was employed (Goloboff et al. 2008) with searches by “new technology”, search level 50, sectorial searches included, tree drift, and tree fusing (Goloboff 1999), hitting the best length 100 times. Parsimony Jackknife absolute frequencies (Farris et al. 1996) were also estimated using “new technology” and requesting 10 hits, driven searches, and 1000 replicates. Edition of trees were made with FIGTREE (Rambaut 2014).

## Results

### Aplastodiscus lutzorum sp. n.

Taxon classificationAnimaliaAnuraHylidae

http://zoobank.org/C506C42B-20FF-41B6-9E5F-177E50C3415F

Figs 1 , 2

Aplastodiscus perviridis Garcia et al. (2001)
Aplastodiscus perviridis Bastos et al. (2003)
Aplastodiscus perviridis Morais et al. (2012)
Aplastodiscus perviridis Valdujo et al. (2012)
Aplastodiscus sp. Santoro and Brandão (2014)
Aplastodiscus sp. 3 Berneck et al. (2016)

#### Holotype.

**Figure 1.**
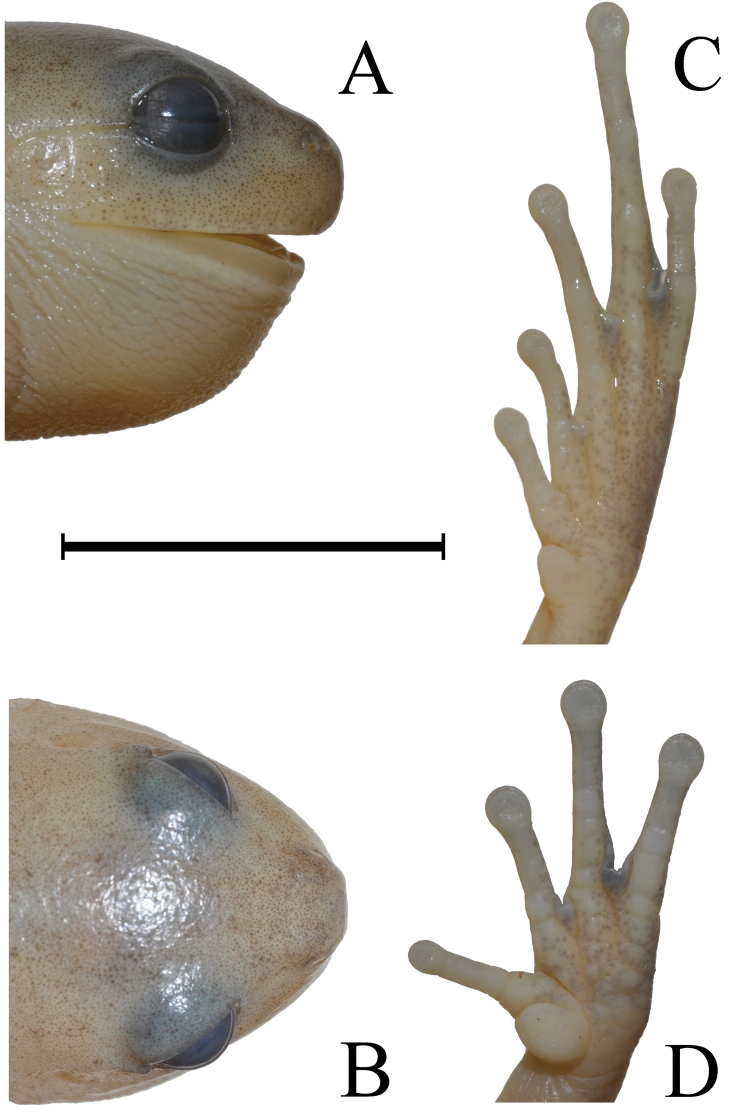
Holotype of *Aplastodiscus lutzorum* sp. n. (AAG-UFU 864). **A** Lateral view of head **B** dorsal view of head **C** plantar view **D** palmar view. Scale bar 12 mm.

**Figure 2.**
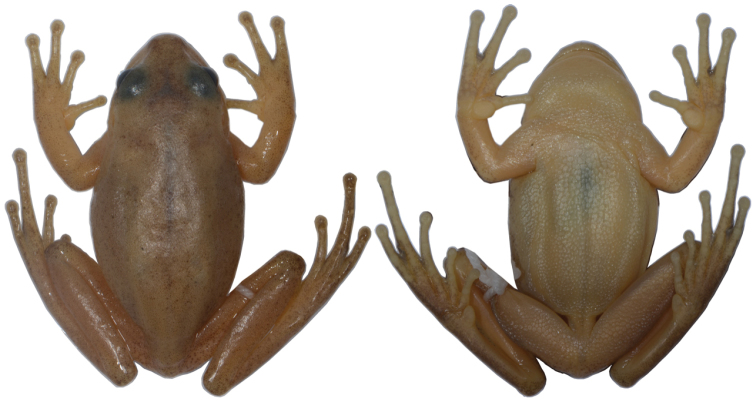
Dorsal and ventral views of the Holotype of *Aplastodiscus lutzorum* sp. n. (AAG-UFU 864).

(Figs 1 and 2) AAG-UFU 864. Adult male collected at Fazenda São Bento (14°09'39"S, 47°34'55"W; 1150 meters above sea level), Municipality of Alto Paraíso de Goiás, Goiás State, Brazil, on 12 December 2011, by A. A. Giaretta and K. G. Facure.

#### Paratypes.

CFBH 22777–80, four adult males collected at Fazenda Água Limpa, Brasília, Distrito Federal (15°56'55.45"S; 47°56'17.83"W) on 18 February 2009. AAG-UFU 863, 865-867 and AAG-UFU 1639 collected on December 2012, AAG-UFU 3343 on 29 November 2013, AAG-UFU 3350–51, 5073–76, 5091, AAG-UFU 0867, adult female, all collected with the holotype. CHUNB 17015–17016 adult males collected on 31 December 1995 at Alexânia, Goiás (16°5'42.00"S; 48°31'20.60"W), CHUNB 17018, adult male collected on 12 December 1985 at Alexânia, Goiás, and CHUNB 74504–74508 adult males from Fazenda Água Limpa, Brasília, Distrito Federal, collected on March 2013. All localities are in Brazil.

#### Referred specimens.

All males. MCN-AM 8809–12 and 8767–72 from AHE Queimado, Unaí, Minas Gerais (16°20'55.51"S; 46°52'48.93"W), collected on February–March 2007.

#### Diagnosis.

*Aplastodiscus lutzorum* sp. n. belongs to the *Aplastodiscus perviridis* Group and thus bears bicolored irises, lacks webbing between toes I and II, has reduced webbing among the remaining toes, and lacks peri-cloacal ornamentation. The new species is diagnosed by its small SVL for the *Aplastodiscus perviridis* Group (30–36 mm) and by the advertisement call 2.5 times longer.

#### Description of holotype.

Adult male: head 20% wider than long; snout rounded in profile, nearly rounded in dorsal view; *canthus rostralis* curved; loreal region concave; nostrils ovoid; internarial region grooved. Supratympanic fold distinct, from posterior corner of orbit to insertion of forearm; tympanum distinct, almost circular; tympanum diameter 48.5% of eye diameter. Upper eyelid smooth as the dorsum. Thoracic fold just discernible. External vocal sac single, subgular, and expanded. Fingers long, slender, no lateral fringe, bearing discret round terminal discs; relative lengths of fingers I, II, IV, III; similar sized discs on fingers II, III and IV, larger than disc of Finger I. Diameter of disc of Finger III equals to diameter of Toe IV, about 42% eye diameter. Subarticular tubercles well defined, rounded; supernumerary tubercles poorly defined on palm, rounded; inner metacarpal tubercle large, rounded, about four times the size of subarticular tubercles; other metacarpal tubercle barely defined; spine of prepollex absent; no nuptial pads; ulnar crest barely defined. Hand webbing formulae: I-II 2--3- III 2^+^-2 IV. Tarsal texture smooth; tarsal fold discrete, extending to the entire length of tarsus; heel smooth; inner metatarsal tubercles large, elongate, three times the size of foot subarticular tubercles; outer metatarsal tubercle absent; subarticular tubercles distinct, large, and rounded, diameter about 3/4 width of terminal disc on the same toe; supernumerary tubercles absent; toes long, slender, without lateral fringe; toes bearing rounded discs, smaller in diameter to those of fingers II-IV. Foot webbing formula: I 2^+^ - 3- II 2^+^ - 3^1/2^ III 2^+^ - 4- IV 3^+^ - 2V. Supra cloacal fold absent. Skin on dorsum smooth; skin on throat, belly, ventral surface of thigh, and arm granular. Dorsal and dorsolateral surfaces almost entirely pale yellow with small dark spots or mottles on dorsal surfaces. Belly pale yellow. Measurements of the holotype (mm): SVL 34.6, HL 10.6, HW 11.4, ED 3.3, TD 2.1, END 3.2, IOD 5.3, THL 18.1, TBL 15.8, and FL 18.4 (Table 2).

**Table 2.** Measurements (in millimeters) of the type-series of *Aplastodiscus lutzorum* sp. n. Abbreviations are: SVL (snout-vent length), HL (head length), HW (head width), ED (eye diameter), TD (tympanum diameter), END (eye-nostril distance), IOD (interocular distance), THL (thigh length), TBL (tibia length), and FL (foot length).

Measurement	Holotype	Female paratype	Males paratypes N = 25 (Mean)
SVL	34.6	33.7	30.7–36 (33.5)
HL	10.6	11.4	8.8–11.4 (10.5)
HW	11.4	11.1	10.5–12.4 (11.4)
ED	3.3	3.4	3–3.7 (3.4)
TD	2.1	2.4	1.5–2.4 (2)
END	3.2	3.1	1.6–3.3 (2.7)
IOD	5.3	5.7	4.5–5.9 (5.4)
THL	18.1	16.2	12–18.7 (17)
TBL	15.8	16.4	14.2–18.5 (16)
FL	18.3	18.8	14.9–19.6 (17)

Color in life of the type-series: Dorsal head surface dark green, almost olive. Dorsal body surface and flanks yellowish green with small and scattered melanophores. The superior third of eye is golden, whereas the inferior 2/3 is red copper. Eye surrounded by a black ring. Vocal sac bluish green. In preservative, colors fade to pale beige and the dorsum shows several dark brown dots, making it darker than other parts of the body. The belly is uniformly pale yellow.

#### Variation in the type series.

The main variation in type series is the body size (Table 2). Small brown chromatophores are along the dorsal skin; but the amount of these chromatophores is variable, ranging from sparse to dense.

#### Calls.

**Figure 3.**
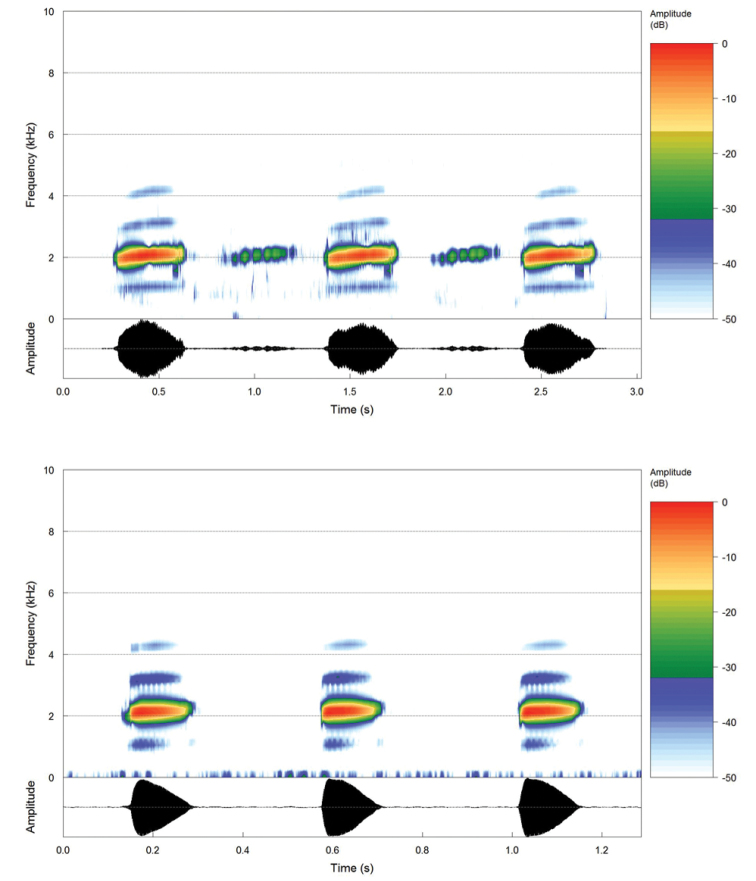
Above, audiospectrogram and oscillogram of three advertisement calls of the holotype of *Aplastodiscus lutzorum* sp. n. (Chapada dos Veadeiros, 12 December 2011, air temperature 20 °C); the background calls are from another male calling in antiphony. Bellow, audiospectrogram and oscillogram of three advertisement call of *Aplastodiscus perviridis* (Serra da Bocaina, 10, January 2012, air temperature 16 °C).

**Figure 4.**
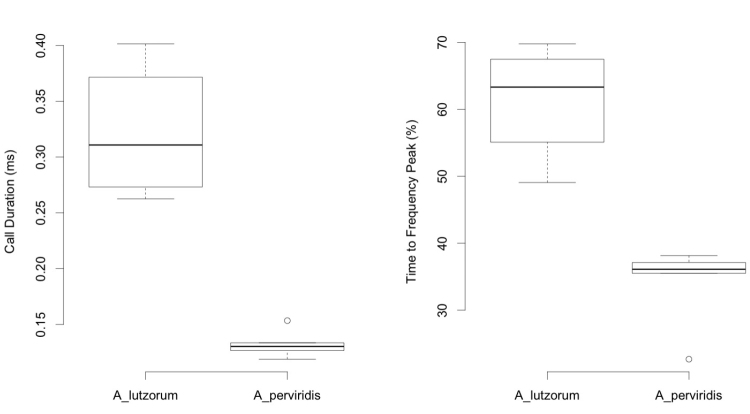
A comparison between duration and frequency peak time of *Aplastodiscus lutzorum* (N = 12 males) and *Aplastodiscus perviridis* (N = 5 males). In both samples, calls are of topotypes.

Advertisement calls of *Aplastodiscus lutzorum* sp. n. (Figure 3 and 4, Table 1) are long regularly-spaced single notes released at a mean rate of 39 calls/minute (SD = 8; n = 12 males). Calls resemble a whistle lasting around 0.26–0.40s. Most of the energy is between 1,494–1,732 Hz and 2,334–2,647 Hz, peaking between 1,884–2,156 Hz. Call exhibits an ascending frequency modulation, reaching its maximum between 49–70% of the call duration.

*Aplastodiscus perviridis*’ advertisement call (Figure 3 and 4, Table 1) is released in groups of 1–11 (mode = 5–7); within groups, call released at a rate of 140/min. Calls resemble a short whistle lasting around 0.12–0.15 s. Most of the energy is between 1,587–1,806 Hz and 2,419–2,750 Hz, peaking between 1,981–2,153 Hz. Call with an ascending frequency modulation, reaching its maximum between 23–38% of the call duration. The advertisement call of *Aplastodiscus cochranae* is described by Garcia et al. (2001) as being barely indistinguishable from the call of *Aplastodiscus perviridis*.

#### Natural history and geographic distribution.

**Figure 5.**
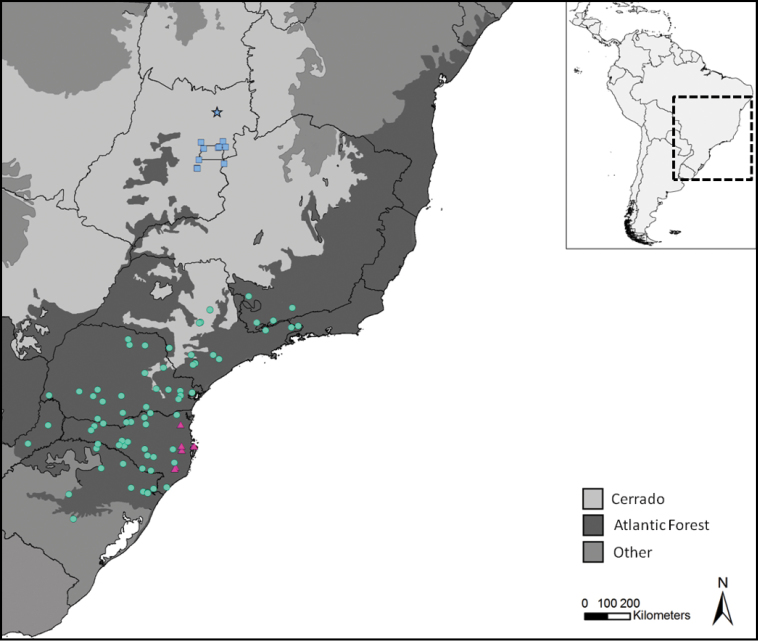
Geographic distribution of *Aplastodiscus cochranae* (pink triangles), *Aplastodiscus perviridis* (green circle), and *Aplastodiscus lutzorum* sp. n. (blue squares, blue star indicates its type-locality). Note that *Aplastodiscus lutzorum* shows a disjunctive distribution regarding the other *Aplastodiscus* species, occurring deep within Cerrado Biome.

**Figure 6.**
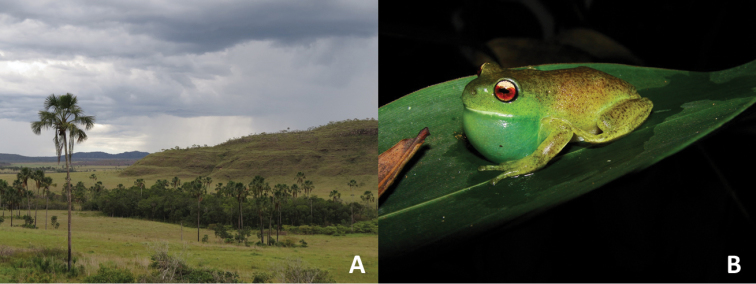
**A** The habitat of *Aplastodiscus lutzorum* sp. n.: flooded gallery forests **B** A male in calling activity at Fazenda Água Limpa, Brasília, Distrito Federal, Brazil.

All specimens of *Aplastodiscus lutzorum* sp. n. were collected along gallery forests with scattered buriti (*Mauritia flexuosa*) palm trees within the Cerrado Biome (see also Brandão and Araujo 2002; Morais et al. 2012; Santoro and Brandão 2014) (Figure 5). A female bearing large oocytes seen by the transparency of the body walls was found in mid-December and calling males were found from December to March. Males call during the night in proximity of riverine forests (< 2m), perched on leaves or branches from the water level to 5 m high (Figure 6). *Aplastodiscus perviridis* males call during the night along swamps in open areas, perched on grass leaves or bushes bordering streamlets, from 0.5 m to 3 m high. Tadpoles are unknown. Sometimes, the species also uses places with wet and soft mud soil, covered by a layer of dense bush, in places where the forest was removed, but is under secondary growth. Sympatric frog species were *Hypsiboas ericae* (Caramaschi and Cruz 2000) and *Hypsiboas albopunctatus* (Spix, 1824). All localities where *Aplastodiscus lutzorum* sp. n. was found are 1000 m above sea level or more.

#### Etymology.

The new species is named after the Brazilian scientists Adolfo and Bertha Lutz, who were pioneers in discovering and studying *Aplastodiscus* and some species of *Hyla* now included in the genus *Aplastodiscus*.

#### Comparison with congeneric species.

*Aplastodiscus lutzorum* sp. n. can be distinguished from the species of other groups of *Aplastodiscus* (*Aplastodiscus albosignatus*, *Aplastodiscus albofrenatus*, and *Aplastodiscus sibilatus* groups) by the presence of bicolored irises, the lack of the webbing between toes I and II, the webbing among the remaining toes reduced, and the absence of peri-cloacal ornamentation. The new species is diagnosed from *Aplastodiscus perviridis* and *Aplastodiscus cochranae* by having smaller SVL (30–36 mm SVL in the new species, 38–44.7 mm in *Aplastodiscus perviridis*, and 41–46.4 mm in *Aplastodiscus cochranae*) and longer advertisement calls (0.38–0.42 in new species, 0.14–0.17 in *Aplastodiscus perviridis* and 0.10–0.18 in *Aplastodiscus cochranae*). From *Aplastodiscus cochranae* it can be also distinguished by the green dorsal color in life (*Aplastodiscus cochranae* is brown) and by the absence of a brown line on the loreal region and a white line in the cloacal region (both present in *Aplastodiscus cochranae*). (Figures 1–4; Tables 1 and 2).

#### Phylogenetic relationships and genetic p-distances.

**Figure 7.**
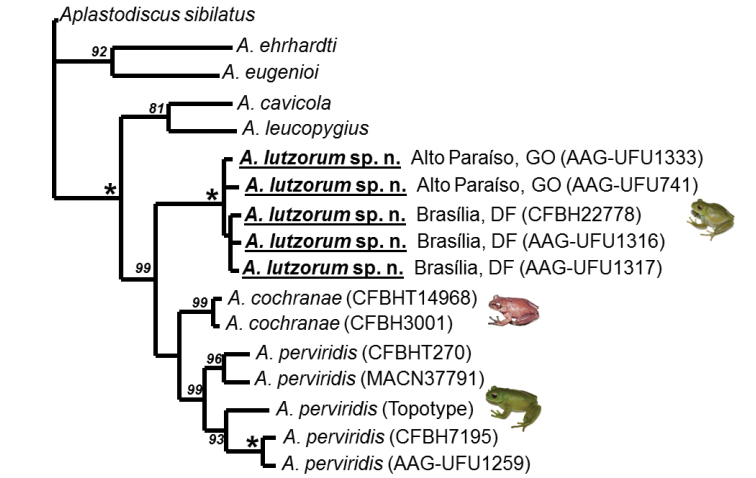
One of the four most parsimonious trees recovered. Asterisks indicate 100% Parsimony Jackknife absolute frequency; only values above 75% are shown. See Suppl. material 2 for details and complete locality names.

The two DNA fragments aligned and concatenated resulted in a matrix of 1,227pb. Our analysis recovered four most parsimonious trees with 808 steps each (Figure 7). The differences in topology among these trees are the position of *Aplastodiscus lutzorum* sp. n. specimens from different localities. *Aplastodiscus lutzorum* sp. n. were recovered as a monophyletic group with 100% Parsimony Jackknife Support (hereafter PJS) being the sister species of *Aplastodiscus perviridis* plus *Aplastodiscus cochranae*. The *Aplastodiscus perviridis* plus *Aplastodiscus cochranae* clade is low supported (54% PJS) and both species are supported by 99% of PJS each. The p-distances calculated for 16s of species in the *Aplastodiscus perviridis* Group range from zero to 5.9% (for all distances see Table 3). The internal distances among specimens of the new species range from zero to 0.93%. The p-distance in 16s between the new species and *Aplastodiscus perviridis* is 4.4–5.8% and between the new species and *Aplastodiscus cochranae* is 4.0–4.5%.

**Table 3.** Uncorrected pairwise sequence distances (p-distances) of the Cytochrome c oxidase subunit 1 (above the diagonal) and 16s fragments (below the diagonal) for the species of the *Aplastodiscus perviridis* species Group. See the Material and methods section for the primers that delimitate each fragment. Values are in percentage.

		1	2	3	4	5	6	7	8	9	10	11	12
**1**	*Aplastodiscus cochranae* CFBH3001 Rancho Queimado, SC	—	1.06	9.13	–	–	9.55	8.92	9.77	9.98	9.77	8.92	10.19
**2**	*Aplastodiscus cochranae* CFBHT14968 Lauro Muller, SC	–	—	9.34	–	–	9.77	9.34	9.98	10.19	9.98	9.13	10.4
**3**	*Aplastodiscus lutzorum* sp. n. CFBH22778 Brasília, DF	4.28	–	—	–	–	0.42	0.42	10.4	9.34	9.98	9.13	9.55
**4**	*Aplastodiscus lutzorum* sp. n. AAG1316 Brasília, DF	4.28	–	0.56	—	–	–	–	–	–	–	–	–
**5**	*Aplastodiscus lutzorum* sp. n. AAG1317 Brasília, DF	4.09	–	0.37	0.19	—	–	–	–	–	–	–	–
**6**	*Aplastodiscus lutzorum* sp. n. AAG1333 Alto Paraíso, GO	4.09	–	0.37	0.19	0	—	0.85	10.19	9.13	9.77	8.92	9.55
**7**	*Aplastodiscus lutzorum* sp. n. AAG741 Alto Paraíso, GO	4.46	–	0.93	0.19	0.56	0.56	—	10.4	9.34	9.98	9.13	9.55
**8**	*Aplastodiscus perviridis* CFBH18119 Topotype	3.35	–	5.2	5.2	5.02	5.02	5.58	—	5.3	6.37	4.03	5.1
**9**	*Aplastodiscus perviridis* CFBH7195 Santo Antônio do Pinhal, SP	3.16	–	5.39	5.39	5.2	5.2	5.76	2.23	—	6.16	4.25	0.64
**10**	*Aplastodiscus perviridis* CFBHT270 São Bento do Sul, SC	2.23	–	4.83	4.83	4.65	4.65	5.02	2.79	2.42	—	2.76	5.94
**11**	*Aplastodiscus perviridis* MACN37791 Misiones, Argentina	3.16	–	5.76	5.76	5.58	5.58	5.95	3.35	3.16	0.93	—	4.03
**12**	*Aplastodiscus perviridis* AAG1259 Atibaia, SP	2.97	–	5.39	5.39	5.2	5.2	5.76	2.23	0.37	2.42	2.97	—

## Discussion

The *Aplastodiscus perviridis* Group now includes a third species, *Aplastodiscus lutzorum*, a species diagnosed mainly by its advertisement call, small size, and genetic differentiation. Genetic p-distances and phylogenetic topology support our hypothesis of the new species. Garcia et al. (2001), when re-describing *Aplastodiscus perviridis*, included six specimens that here we recognize as *Aplastodiscus lutzorum* (CHUNB 404; 268–70; 1378; 1704) the minimum snout-vent lengths values of *Aplastodiscus pervirids* in that work overlaps the SVL of the new species. Garcia et al. (2001) also discuss an unusual condition for any anuran species, observed in *Aplastodiscus perviridis* and *Aplastodiscus cochranae*: identical advertisement calls with clearly distinct coloration (*Aplastodiscus cochranae* is the only brown species of the genus). The description of the advertisement calls of *Aplastodiscus perviridis* in Garcia et al. (2001) were based on specimens from Ribeirão Branco (São Paulo State) and so, do not belong to *Aplastodiscus lutzorum*. The identical advertisement call shared by *Aplastodiscus perviridis* and *Aplastodiscus cochranae* highlight the taxonomic importance of the differences we found in *Aplastodiscus lutzorum*.

Berneck et al. (2016) included only one specimen of the *Aplastodiscus lutzorum* (as *Aplastodiscus* sp. 3), therefore the monophyly of the new species was tested for the first time by our analysis. Berneck et al. (2016) recovered *Aplastodiscus lutzorum* as a sister species of *Aplastodiscus cochranae*, a topology not corroborated by the present work, where the new species is a sister species of *Aplastodiscus cochranae* plus *Aplastodiscus perviridis*. In the present work, the node of *Aplastodiscus perviridis* plus *Aplastodiscus cochranae* is supported by less than 70% of PJS while in Berneck et al. (2016) the node of *Aplastodiscus lutzorum* plus *Aplastodiscus cochrane* was poorly supported (also less than 70%). Those are possibly the reason of the instability in the internal relationships of members of the *Aplastodiscus perviridis* Group. However, our dataset and taxon sampling is very reduced in relation to that of Berneck et al. (2016) and so the analysis of these authors is preferable for relationships of *Aplastodiscus* species.

The scope of this paper was not to test biogeographic hypotheses. However, *Aplastodiscus lutzorum* is the only species of *Aplastodiscus* that occurs deep in the Cerrado Biome (see Silva et al. 2006) with a disjunctive distribution from its sister species of the Atlantic Forest (Valdujo et al. 2012). Therefore, it seems interesting to point out some remarks on its geographic distribution pattern (Figure 5). The new species has been reported in several localities in the Brazilian Central Plateau and our topology suggests an origin of the *Aplastodiscus perviridis* Group in the Brazilian Central Plateau (Figure 7). However, the low PJF support of the clade *Aplastodiscus perviridis* + *Aplastodiscus cochranae* and the incongruence between our topology and that of Berneck et al. (2016) make any further inference premature.

A population from the dam of Queimado in the municipality of Unaí, in the state of Minas Gerais, Brazil (an area flooded by the construction of a hydroelectric station) was included as “referred specimens” for *Aplastodiscus lutzorum* The conservation status of this population is unknown. We consider the new species to be listed as a “Least Concern”, due to the fact that most of its area of occurrence is in protected places, such as the Parque Nacional da Chapada dos Veadeiros, Área de Relevante Interesse Ecológico (ARIE) do Capetinga/Taquara (Fazenda Água Limpa), Estação Ecológica de Águas Emendadas, and Floresta Nacional de Silvânia.

Goin (1961) suggested that *Chorophilus cuzcanus* Cope, 1878 should be an *Aplastodiscus*, but had not stated that it was *Aplastodiscus perviridis* (as pointed out by Frost 2016). Lutz (1968) suggested that *Chorophilus cuzcanus* was possibly a second species of *Aplastodiscus* at that time. According to Frost (2016), the combination *Chorophilus cuzcanus* is a junior synonym to both *Aplastodiscus perviridis* and *Gastrotheca marsupiata* (Duméril and Bibron 1841). We recognize only the synonym of Duellman and Fritts (1972) for *Gastrotheca marsupiata* as valid; therefore *Aplastodiscus perviridis* has no junior synonyms.

## Supplementary Material

XML Treatment for Aplastodiscus lutzorum

## Supplementary materials

Supplementary material 1 Analyzed sound files; from Ariovaldo A. Giaretta collection Data type: species data This dataset is made available under the Open Database License (http://opendatacommons.org/licenses/odbl/1.0/). The Open Database License (ODbL) is a license agreement intended to allow users to freely share, modify, and use this Dataset while maintaining this same freedom for others, provided that the original source and author(s) are credited.Bianca V. M. Berneck, Ariovaldo A. Giaretta, Reuber A. Brandão, Carlos A. G. Cruz, Célio F. B. Haddad

Supplementary material 2 List of voucher specimens, GenBank accession numbers, and locality data Data type: species data This dataset is made available under the Open Database License (http://opendatacommons.org/licenses/odbl/1.0/). The Open Database License (ODbL) is a license agreement intended to allow users to freely share, modify, and use this Dataset while maintaining this same freedom for others, provided that the original source and author(s) are credited.Bianca V. M. Berneck, Ariovaldo A. Giaretta, Reuber A. Brandão, Carlos A. G. Cruz, Célio F. B. Haddad
