# Supplementary material for: The first species of Aplastodiscus endemic to the Brazilian Cerrado (Anura, Hylidae)
Source: Zookeys. 2017 Jan 3;(642):115–30. doi: 10.3897/zookeys.642.10401 (PMC5240532; doi:10.3897/zookeys.642.10401)
Supplement: Supplementary material 1 — Analyzed sound files; from Ariovaldo A. Giaretta collection [file zookeys-642-115-s001.pdf]

Appendix 1 – Analyzed sound files; from Ariovaldo A. Giaretta collection.
Aplast_spBrasiliaDF1aAAGm671.wav
Aplast_spBrasiliaDF1bAAGm671.wav
Aplast_spBrasiliaDF2aCSB_AAGm671.wav
Aplast_spBrasiliaDF2bCSB_AAGm671.wav
Aplast_spBrasiliaDF2cCSB_AAGm671.wav
Aplast_spBrasiliaDF2dCSB_AAGm671.wav
Aplast_spBrasiliaDF2eCSB_AAGm671.wav
Aplast_spBrasiliaDF3aAAGm671.wav
Aplast_spBrasiliaDF3bAAGm671.wav
Aplast_spBrasiliaDF3cAAGm671.wav
Aplast_spBrasiliaDF4aAAGm671.wav
Aplast_spBrasiliaDF4bAAGm671.wav
Aplast_spBrasiliaDF5aCBS_AAGm671.wav
Aplast_spBrasiliaDF5bCBS_AAGm671.wav
Aplast_spBrasiliaDF6aCBS_AAGm671.wav
Aplast_spBrasiliaDF6bCBS_AAGm671.wav
Aplast_spVeadeirosGO1aAAGm671.wav
Aplast_spVeadeirosGO1bAAGm671.wav
Aplast_spVeadeirosGO2aAAGm671.wav
Aplast_spVeadeirosGO2bAAGm671.wav
Aplast_spVeadeirosGO2cAAGm671.wav
Aplast_spVeadeirosGO3aAAGm671.wav
Aplast_spVeadeirosGO3bAAGm671.wav
Aplast_spVeadeirosGO4aAAGm671.wav
Aplast_spVeadeirosGO4bAAGm671.wav
Aplast_spVeadeirosGO5aAAGm671.wav
Aplast_spVeadeirosGO6aAAGm671.wav
Aplast_spVeadeirosGO6bAAGm671.wav
Aplast_spVeadeirosGO6cAAGm671.wav
Aplast_spVeadeirosGO6dAAGm671.wav
Aperviridis CFBHaddad Bocaina jan09.wav
Aperviridis MGarey Bocaina Out08 indiv1.wav
Aperviridis MGarey Bocaina Out08 indiv2.wav
