# Supplementary material for: The first species of Aplastodiscus endemic to the Brazilian Cerrado (Anura, Hylidae)
Source: Zookeys. 2017 Jan 3;(642):115–30. doi: 10.3897/zookeys.642.10401 (PMC5240532; doi:10.3897/zookeys.642.10401)
Supplement: Supplementary material 2 — List of voucher specimens, GenBank accession numbers, and locality data [file zookeys-642-115-s002.pdf]

Appendix 2. List of vouchers, GenBank accession numbers, and locality data.In bold, sequences produced by the presente work; all others were produced by Faivovich et al. (2005) and Berneck et al. (2016).

**Species**	**Voucher**	**Locality data**	**val-16s (Fragment)**	**Cytochrome oxidase I**
Aplastodiscus cochranae	CFBH3001	Rancho Queimado, Santa Catarina, Brazil	AY843568	KU184043
Aplastodiscus cochranae	CFBHT14968	Lauro Muller, Santa Catarina, Brazil	KU184019	
Aplastodiscus lutzorum sp. nov.	CFBH22778	Brasília, Distrito Federal, Brazil	KU184003	KU184054
Aplastodiscus lutzorum sp. nov.	AAG1316	Brasília, Distrito Federal, Brazil	**KY213875**	**KY213881**
Aplastodiscus lutzorum sp. nov.	AAG1317	Brasília, Distrito Federal, Brazil	**KY213876**	**KY213878**
Aplastodiscus lutzorum sp. nov.	AAG1333	Alto Paraíso, Goiás, Brazil	**KY213877**	
Aplastodiscus lutzorum sp. nov.	AAG741	Alto Paraíso, Goiás, Brazil	**KY213873**	**KY213879**
Aplastodiscus perviridis	CFBH18119	São José do Barreiro, São Paulo Brazil	KU184008	KU184045
Aplastodiscus perviridis	CFBH7195	Santo Antônio do Pinhal, São Paulo, Brazil	KU184016	KU184049
Aplastodiscus perviridis	CFBHT270	São Bento do Sul, Santa Caratina, Brazil	KU184020	KU184057
Aplastodiscus perviridis	MACN37791	Misiones, Argentina	AY843569	KU184041
Aplastodiscus perviridis	AAG1259	Atibaia, São Paulo, Brazil	**KY213874**	**KY213880**
Aplastodiscus ehrhardti	CFBHT11191	São José dos Pinhais, Paraná, Brazil	KU184017	KU184050
Aplastodiscus eugenioi	CFBH17497	São Sebastião, São Paulo, Brazil	KU184007	KU184051
Aplastodiscus cavicola	FSFL848	Congonhas, Minas Gerais, Brazil	KU184023	KU184044
Aplastodiscus sibilatus	CFBH32528	Ibirapitanga, Bahia, Brazil	KU184014	
